# Telemedicine for chronic pain management in rural populations: a narrative review

**DOI:** 10.3389/fpain.2026.1907994

**Published:** 2026-10-13

**Authors:** Jamal Hasoon, Cyrus Yazdi, Christopher L. Robinson

**Affiliations:** 1 Department of Anesthesiology, Critical Care and Pain Medicine, The University of Texas Health Science Center at Houston, Houston, TX, United States; 2 Department of Anesthesiology, Critical Care and Pain Medicine, Beth Israel Deaconess Medical Center, Boston, MA, United States; 3 Department of Anesthesiology and Critical Care Medicine, The Johns Hopkins University School of Medicine, Baltimore, MD, United States

**Keywords:** chronic pain, health disparities, pain management, rural health, telehealth, telemedicine

## Abstract

Chronic pain is among the most prevalent and burdensome health conditions in the United States, disproportionately affecting rural populations who face compounding barriers to specialty care, including geographic distance, transportation burden, specialist shortages, and limited access to multidisciplinary services. These structural inequities contribute to persistent disparities in pain prevalence, pain-related disability, and opioid utilization. Telemedicine is a promising strategy for reducing this access gap, with adoption accelerating markedly following the COVID-19 pandemic. This narrative review examines telemedicine for chronic pain management in rural populations across synchronous video visits, telephone-based encounters, asynchronous electronic consultations, telerehabilitation, tele-delivered cognitive behavioral therapy, and mobile health applications. The evidence base is strongest for remotely delivered cognitive behavioral therapy and selected telerehabilitation interventions, whereas evidence for specialty video programs, electronic consultations, Veterans Health Administration programs, and mobile health remains more heterogeneous and frequently observational. Telemedicine can improve access, psychological outcomes, functional status, and patient convenience while reducing travel burden, but it does not consistently improve pain intensity and may worsen inequities when broadband, device access, digital literacy, or privacy are inadequate. Important implementation challenges include limitations of the remote physical examination, variable patient preferences, technology anxiety, and regulatory uncertainty governing telemedicine prescribing of controlled substances. A hybrid model that uses structured triage, low-bandwidth alternatives, technology-navigation support, standardized opioid monitoring, and clearly defined triggers for in-person evaluation represents the most pragmatic framework for rural chronic pain care in the U.S. context.

## Introduction

Chronic pain remains a prevalent and disabling health condition worldwide. It can cause significant suffering and lead to reduced quality of life, healthcare expenditures, and lost productivity (1–4). Chronic pain encompasses a broad spectrum of conditions including low back pain, osteoarthritis, neuropathic pain, post-surgical pain syndromes, cancer-related pain, and widespread nociplastic pain disorders such as fibromyalgia (5). Effective management often requires a multidisciplinary approach involving medical, psychological, rehabilitative, and interventional therapies.

Despite advances in pain medicine therapies, substantial disparities in access to care persist. Rural populations face particular challenges in obtaining specialized pain management services (6, 7). In many regions, specialists are concentrated in urban academic centers, leaving rural communities underserved. Patients may travel several hours to access care, resulting in delays in diagnosis, fragmented treatment, increased healthcare costs, and poor outcomes (8).

Telemedicine has expanded dramatically and serves as a potential solution to bridge geographic barriers and improve access to specialty care. Although telemedicine utilization increased steadily during the early 2000s, the COVID-19 pandemic accelerated adoption across virtually all medical specialties (9–14). The objective of this narrative review is to examine the role of telemedicine in chronic pain management among rural populations, summarize current evidence, identify implementation challenges, and discuss future opportunities for improving pain care delivery.

## Methods

This narrative review was informed by searches of PubMed/MEDLINE and Google Scholar for English-language publications from January 2010 through July 2026. Search concepts combined terms related to chronic pain, rural health, telemedicine or telehealth, video visits, telephone care, electronic consultations, telerehabilitation, cognitive behavioral therapy, mobile health, and controlled-substance prescribing. We prioritized systematic reviews, randomized trials, large observational studies, program evaluations, and qualitative studies addressing adults with chronic pain in rural or geographically underserved settings. Pediatric-only studies, reports limited to acute postoperative pain, and studies without a meaningful telemedicine or chronic pain component were excluded. Narrative reviews synthesize and contextualize heterogeneous literature without requiring the exhaustive study identification, formal risk-of-bias assessment, or quantitative pooling expected of a systematic review. This approach was selected because the objective was to describe how telemedicine fits within the broader delivery of chronic pain care in rural populations across multiple modalities, outcomes, and implementation settings rather than to estimate a single pooled treatment effect. After the searches described above, all authors reviewed the identified literature for relevance to the review objectives. Findings were analyzed qualitatively and organized according to telemedicine modality, clinical application, rural relevance, study design, and consistency of reported outcomes.

### Rural disparities in chronic pain management

Chronic pain disproportionately affects individuals residing in rural and geographically underserved regions. Compared with urban populations, rural residents often experience higher rates of chronic pain, pain-related disability, and opioid utilization (6, 7, 15, 16). Several demographic, occupational, and socioeconomic factors contribute to this disparity. Rural communities tend to have older populations, greater participation in physically demanding occupations such as agriculture, construction, and manual labor, and higher rates of obesity and tobacco use (15–17). These factors increase the risk of developing chronic musculoskeletal and neuropathic pain conditions (18, 19). Furthermore, rural populations often have reduced access to preventive healthcare services and early interventions, which may contribute to delayed diagnosis and progression of painful conditions (20–22). Collectively, these factors create a disproportionate burden of chronic pain that negatively impacts quality of life, functional status, and overall health outcomes in rural communities.

In addition to a greater burden of disease, rural populations face substantial barriers to accessing specialized pain management services. Pain medicine physicians and multidisciplinary pain programs are concentrated primarily in urban and academic medical centers, resulting in significant geographic disparities in care (23, 24). Patients living in rural areas frequently travel long distances to attend specialty appointments, often incurring transportation expenses, lost wages, and missed workdays (8, 25–27). These barriers may contribute to delayed evaluation, interruptions in treatment, and reduced adherence to recommended therapies (25, 28). Limited availability of physical therapy, behavioral health services, and other multidisciplinary resources further compounds these challenges (29, 30). Consequently, rural patients often rely heavily on primary care providers to manage complex chronic pain conditions despite limited access to specialty consultation and comprehensive pain management resources.

The cumulative effect of these barriers contributes to persistent disparities in pain outcomes and healthcare utilization among rural populations (Table 1).

**Table 1:** Factors contributing to rural disparities in chronic pain management.

Domain	Rural Challenge	Impact on Chronic Pain Care
Demographics	Older average population age	Increased prevalence of degenerative musculoskeletal disorders and chronic pain
Opioid Use	Higher rates of opioid prescribing and long-term opioid exposure in some rural populations	May reflect reduced access to nonpharmacologic and specialty pain care and reinforces the need for multimodal treatment options
Occupation	Higher rates of agricultural, industrial, and manual labor occupations	Greater risk of work-related injuries and chronic musculoskeletal pain
Health Behaviors	Increased prevalence of obesity and tobacco use	Higher risk of pain chronification and associated comorbidities
Preventive Care	Reduced access to preventive and primary healthcare services	Delayed diagnosis and treatment of pain conditions
Specialist Availability	Limited number of pain medicine specialists	Delayed referrals and prolonged wait times
Geographic Barriers	Long travel distances to specialty clinics	Reduced access to ongoing care and follow-up
Multidisciplinary Services	Limited access to physical therapy, psychology, and rehabilitation programs	Reduced availability of comprehensive pain management

Table 1. Factors contributing to disparities in chronic pain prevalence, access to specialty care, and treatment outcomes among rural populations. These challenges highlight potential areas where telemedicine and digital health interventions may improve access to comprehensive pain management services.

### Telemedicine modalities in chronic pain management

Telemedicine encompasses synchronous video visits, audio-only care, asynchronous electronic consultations, remote patient monitoring, and mobile health applications (31, 32). The appropriate modality depends on diagnostic certainty, the need for a hands-on examination, patient digital access, and the goals of the encounter. Video permits observation of affect, gait, gross motor function, and functional movement, whereas telephone encounters provide a lower-bandwidth option for straightforward follow-up and care coordination. A 2016 evaluation of a Massachusetts General Hospital telepain service to Martha's Vineyard completed 238 video evaluations over 13 months; among 49 surveyed patients, mean scores for every satisfaction item exceeded 4.3 of 5, indicating a favorable experience (33). In a cross-sectional patient satisfaction survey of 61 chronic pain patients treated at a tertiary university pain center (Bern University Hospital, Switzerland), telemedicine acceptance scores averaged 6.25 out of 10, with higher pain intensity and anxiousness correlating negatively with acceptance. A slight majority of respondents expressed a preference for in-person consultations when given the choice. Notably, patients who had previously experienced telehealth consultations showed greater willingness to continue telemedicine use compared with those without prior exposure, suggesting that familiarity reduces hesitancy and improves long-term acceptability (34). More recent rural-focused evidence also supports flexible modality selection. A 2025 retrospective study of 9,359 rural-dwelling patients found that both video and telephone telehealth remained in use and identified lower video use and patient-portal activation among several groups at risk for digital exclusion, including older adults and some publicly insured, Spanish-speaking, and minoritized patients (35).

Telephone-based consultations remain important for patients who lack reliable broadband, video-capable devices, or confidence navigating telehealth platforms (35, 36). Audio-only encounters are particularly useful for established patients undergoing medication review, brief symptom reassessment, behavioral health follow-up, or care coordination. They are less appropriate for a new undifferentiated pain complaint, progressive neurological symptoms, suspected infection, recent trauma, or other presentations in which direct examination may materially alter diagnosis or treatment.

Asynchronous telemedicine, in which clinical information is transmitted electronically and reviewed by a specialist at a later time, offers additional flexibility for rural pain care. E-consult programs have also been successfully implemented within integrated health systems. The Veterans Health Administration completed over 3.1 million e-consults across 41 specialties between 2012 and 2018, with only 21.5% resulting in an in-person visit within 12 months, suggesting effective triage and reduction of unnecessary referrals. Each e-consult saved patients approximately 84.3 miles of driving, a particularly meaningful benefit for rural veterans (37). The Los Angeles County Department of Health Services similarly adopted e-consults across its safety-net system, enabling specialists to triage urgency and manage clinical questions through electronic dialogue without requiring a face-to-face visit (38).

These telemedicine models enable rural primary care providers to access specialist expertise without the logistical burden imposed by traditional referral pathways, shortening the time from initial pain presentation to receipt of evidence-based management recommendations.

### Evidence supporting telemedicine for chronic pain management

The evidence supporting telemedicine-delivered chronic pain care includes randomized clinical trials, systematic reviews, observational cohorts, health-system program evaluations, and qualitative studies. Randomized evidence is most developed for remotely delivered psychological interventions and selected telerehabilitation programs, whereas specialty video clinics, electronic consultation programs, and many rural implementation models are supported primarily by observational or qualitative data. Outcomes also vary by modality: improvements in access, engagement, psychological function, and travel burden are more consistently reported than large or durable reductions in pain intensity.

### Remote cognitive behavioral therapy for chronic pain

Among the strongest available evidence is the phase 3 pragmatic randomized clinical trial by DeBar et al., published in JAMA in 2025, which enrolled more than 2,000 patients with high-impact chronic musculoskeletal pain from four geographically diverse U.S. health systems (39). Participants were randomized to one of two remote, eight-session cognitive behavioral therapy for chronic pain (CBT-CP) interventions—one delivered by a health coach through telephone or videoconferencing and one completed independently online—or to usual care. Both remote CBT-CP modalities produced clinically meaningful and sustained improvements in pain severity compared with usual care through 12 months (39). At the 3-month primary endpoint, the health coach-led intervention demonstrated the largest effect, although by 6 and 12 months the two active interventions produced comparable benefits (39). These findings are consistent with a Cochrane review concluding that remotely delivered CBT likely reduces pain intensity and anxiety immediately after treatment compared with usual care (40). These findings support remote delivery of structured psychological pain interventions as a scalable, effective solution for closing the rural-urban gap in access to evidence-based nonpharmacologic care. In this trial, usual care consisted of participants continuing their ongoing medical care and receiving a chronic pain resource guide, without access to either of the study CBT-CP programs (39).

### Telerehabilitation for chronic low back pain

Telerehabilitation represents another well-studied avenue for extending pain rehabilitation services to rural populations. Physical therapy is established as cost-effective for improving pain and disability in patients with chronic low back pain (LBP), yet only an estimated 7%–13% of individuals with LBP ultimately receive physical therapy services, with access particularly constrained in rural communities where approximately 40% fewer physical therapists are available per capita compared with metropolitan regions (29). The ARBOR-Telehealth trial is a randomized study enrolling 434 patients with chronic low back pain from rural primary care clinics (29). Participants are randomized to either a telerehabilitation program delivered by trained physical therapists through a web-based video platform or a standardized patient education program. The primary objective is to determine whether telehealth-delivered rehabilitation can reduce low back pain–related disability and opioid utilization compared with patient education alone (29). This study aims to address barriers to accessing specialized physical therapy services in underserved populations. Earlier evidence from a randomized controlled trial of exercise-based telerehabilitation for nonspecific chronic LBP demonstrated comparable therapeutic efficacy to conventional outpatient-based physical therapy, with no significant differences in disability, pain intensity, or fear-avoidance outcomes at 8 weeks (41). A scoping review published in 2024, encompassing 28 studies on exercise-based telerehabilitation for chronic LBP, found substantial heterogeneity in delivery modalities and outcome measures but consistently identified pain and disability as the most commonly assessed and most frequently improved outcomes, with smartphone applications the dominant mode of asynchronous intervention delivery (42). Additional evidence from a longitudinal observational study of telehealth physical therapy using videoconferencing for chronic LBP demonstrated significant improvements in disability, pain intensity, pain interference, and physical function at 10- and 26-week follow-ups, with generally positive patient satisfaction, though only 39.5% perceived the quality as equal to in-person physical therapy (43).

### Veterans health administration telePain programs

The Veterans Health Administration (VHA) has been a major early adopter of telehealth-delivered pain care for rural populations. The TelePain program serving communities in Alaska, Washington, and Oregon provides specialty pain consultation through home-based video visits or telehealth rooms at community outpatient clinics (44). Qualitative evaluation found general satisfaction, appreciation for knowledgeable and supportive clinicians, and substantial value from reduced travel, while also identifying inconsistent follow-up, negative perceptions of behavioral health treatment, and preference for in-person care among some veterans (44). The TelePain-Empower Veterans Program (TelePain-EVP), a nonpharmacological pain management program incorporating Whole Health, Acceptance and Commitment Therapy, and Mindful Movement through interdisciplinary personalized coaching, has been evaluated through both qualitative and quantitative methods (45, 46). This included improvements in veterans’ coping skills, sense of community, and interpersonal relationships, though notably participants did not report self-perceived reductions in pain or medication use (45). There was also a significant decrease in pain catastrophizing from baseline to post-treatment (*p* < 0.001) and improvements in anxiety, depression, and activities engagement, though pain catastrophizing improvements were not sustained at 26-week follow-up (46). Overall, the VHA experience supports benefits for access and psychological outcomes, but robust and durable improvements in pain intensity remain less certain.

### Mobile health applications

Mobile health technologies represent another expanding modality for chronic pain management, with particular relevance to rural populations who face barriers to frequent in-person follow-up. A systematic review published in 2024 encompassing 89 articles and 26,429 participants evaluated 25 years of mobile technology tool use in chronic musculoskeletal pain management, finding that mobile applications were the most commonly employed modality (88% of studies), with usability, feasibility, and acceptability rated as generally high among the subset of studies that assessed these outcomes (26% of included studies) (47). A separate systematic review of 22 RCTs involving 2,641 patients confirmed beneficial effects of mHealth systems on pain intensity, quality of life, and functional disability across multiple chronic pain conditions, including chronic low back pain, chronic neck pain, fibromyalgia, and osteoarthritis (48). Another meta-analysis of digital health interventions for musculoskeletal pain (56 RCTs, 9,359 participants) found small but significant effects on pain, disability, quality of life, emotional functioning, and self-management based on moderate-quality evidence (49).

For rural patients, mobile health applications offer several advantages beyond convenience. Digital pain diaries and symptom tracking platforms enable structured, longitudinal data collection between clinical encounters, providing clinicians with a better understanding of pain patterns than is possible during intermittent office visits. Daily pain data collected via smartphone applications have revealed variability patterns and associations with modifiable variables such as fatigue, mood, and morning stiffness that would be missed during periodic clinical assessments (50). Digital pain mapping tools have also demonstrated the ability to capture fluctuations in pain distribution and intensity over sustained periods through remote momentary assessment (51). Automated medication reminders, patient education modules, and behavioral activation prompts embedded in smartphone applications can extend the reach of multidisciplinary pain interventions into patients’ daily lives without requiring additional provider time (52). Importantly, a longitudinal cohort study of nearly 10,000 patients across the United States demonstrated that a completely remote multimodal digital care program for musculoskeletal pain achieved equivalent clinical outcomes in rural and urban populations, with rural residents showing higher engagement with educational content and higher program completion rates, supporting the potential of digital care programs to promote health equity (53). These findings are encouraging but should not be interpreted to mean that smartphone-dependent programs are universally equitable. Rural older adults, patients with limited broadband, individuals with visual, cognitive, or motor impairments, and those with limited financial resources may be underrepresented in app-based studies or unable to participate at all.

For digitally underserved patients, the most appropriate mobile strategy may be a stepped approach that begins with SMS reminders, telephone coaching, automated voice calls, mailed educational materials linked to brief telephone follow-up, caregiver-assisted participation, or loaner devices rather than requiring a smartphone application (35, 54, 55). Health systems can further reduce barriers by providing a technology navigator who helps patients test equipment, establish portal access, and choose the lowest-bandwidth modality that still meets the clinical goal. Technology anxiety, privacy concerns, language needs, cultural preferences, and reluctance to discuss substance use or mental health from the home should be assessed explicitly rather than treated as patient nonadherence.

### Quality of evidence

Because this is a narrative review, the following categories represent an informal appraisal of relative evidence strength rather than a formal GRADE assessment: Relative strength was judged qualitatively according to study design and hierarchy of evidence, sample size, consistency of findings across studies, directness to chronic pain and rural populations, and duration of follow-up; these labels were not generated using a validated scoring instrument.

Remote psychological therapy—moderate-to-high evidence strength. Support includes a large pragmatic randomized trial and a Cochrane systematic review, with sustained benefit for selected outcomes. Generalizability is reduced for patients unable to access or complete remote programs (39, 40).

Telerehabilitation—moderate evidence strength. Randomized trials, observational cohorts, and reviews generally support improvements in pain and function, but intervention intensity, supervision, technology, and outcome measures are heterogeneous (29, 41–43).

Mobile health—moderate evidence strength. Multiple systematic reviews and randomized trials show small benefits, but app design and engagement vary considerably, rural subgroup analyses are limited, and digital exclusion may bias results toward technologically connected participants (47–55).

Specialty video visits and electronic consultations—low-to-moderate evidence strength. These models consistently improve convenience, triage, and travel burden, but most data are observational, satisfaction-based, or derived from integrated health systems with mature infrastructure (33, 37, 38).

VHA TelePain and related multidisciplinary programs—low-to-moderate evidence strength. Program evaluations and qualitative studies demonstrate feasibility and psychological benefit, but lack of randomized comparisons and inconsistent improvement in pain intensity or medication use limit causal conclusions (44–46).

### Limitations of telemedicine in chronic pain management

While telemedicine offers important advantages for expanding access to chronic pain care, certain inherent limitations must be acknowledged. Physical examination remains a fundamental component of comprehensive pain assessment, and aspects of the hands-on examination cannot be fully replicated through a video screen (10, 13). The American Academy of Physical Medicine and Rehabilitation guideline on telehealth notes that tactile stimuli such as pain with palpation and presence of warmth to suggest infection and manual aspects of the examination such as range of motion of the hip and tests to evaluate for spasticity or rigidity are difficult to perform via telehealth and may require modifications or caregiver assistance (56). However, the evidence is more nuanced than a blanket limitation: studies have found that telehealth examinations have findings similar to those performed in-person for patients with spinal conditions, low back pain, and many extremity assessments, with palpation and range of motion being the primary areas of reduced agreement, particularly in older patients with more comorbidities. However, the evidence is more nuanced than suggesting a universal limitation of telehealth. Studies have demonstrated good agreement between telehealth and in-person examinations for patients with spinal disorders, low back pain, and a variety of extremity conditions. The greatest discrepancies are typically observed in assessments requiring palpation and precise range-of-motion measurements, with lower concordance reported among older patients and those with greater medical complexity and comorbidities (56). For pain-specific assessment, a multidisciplinary consensus panel developed an algorithmic approach to the virtual physical examination for pain practitioners, providing structured guidance for remote musculoskeletal and neurological evaluation. The “Point To the Area of Pain” test has also been described as a clinically useful telehealth technique for focal nociceptive and neuropathic pain, with 88.5% of surveyed pain providers agreeing that it provides clinically relevant information in the absence of a hands-on exam (57). This can easily be performed through virtual visits with video components. Nevertheless, assessment of allodynia, hyperalgesia, and detailed sensory mapping remain substantially limited in the virtual setting, as these require direct tactile stimulation that cannot be reliably self-administered.

For patients with complex presentations requiring interventional procedures such as epidural steroid injections, spinal cord stimulation trials, or nerve blocks telemedicine can facilitate pre-procedural consultation, shared decision-making, and post-procedural follow-up, but cannot substitute for the procedure itself, necessitating continued in-person care for the interventional component of pain management (58, 59). One of telemedicine's most effective uses in chronic pain management has been post-injection follow-up and assessment of further necessary interventions, reducing the need for additional in-person visits for routine post-procedural monitoring (58). Hybrid care models that integrate telehealth visits for longitudinal monitoring and behavioral interventions with scheduled in-person encounters for physical examination and procedures therefore represent the most pragmatic approach for many rural pain patients. Supporting this hybrid approach, a cross-sectional study of persistent pain patients found that a majority of patients preferred videoconferencing while 26% preferred face-to-face attendance, and preferences varied by discipline (60).

Privacy, data security, and patient-centered acceptability represent additional implementation considerations. Patients may lack a private or quiet location for discussion of psychosocial stressors, substance use, mental health symptoms, or functional impairment (61). Safety-net clinicians have reported difficulty monitoring opioid adherence and misuse through telehealth, particularly when patients have unstable housing or limited access to laboratory testing (61). Technology anxiety, language barriers, disability, cultural preferences, and a desire for direct interpersonal contact may further influence engagement. Secure platforms, clear privacy counseling, interpreter access, and patient choice between video, audio-only, and in-person care should therefore be incorporated into program design (62).

### Implementation challenges: regulation, cost, and patient support

Controlled-substance prescribing remains a major source of operational uncertainty for U.S. telemedicine programs. The Ryan Haight Online Pharmacy Consumer Protection Act generally requires at least one in-person medical evaluation before a practitioner prescribes controlled substances through the internet, unless a statutory or regulatory telemedicine exception applies (63). During the COVID-19 public health emergency, federal flexibilities permitted DEA-registered practitioners to prescribe Schedule II-V controlled medications through qualifying telemedicine encounters without a prior in-person evaluation. DEA and HHS have extended these flexibilities through December 31, 2026, while permanent special-registration rules remain under development (63, 64). Because the current authority is temporary and prescriptions must still comply with federal and state law, rural pain programs should maintain current regulatory review, document the modality and clinical justification for controlled-substance prescribing, use prescription drug monitoring and toxicology testing when clinically indicated, and establish timely pathways for in-person evaluation if federal requirements change or the clinical risk exceeds what can be managed remotely.

Cost-effectiveness should be evaluated from both the patient and health-system perspectives. Telemedicine programs require upfront investment in secure platforms, workflow redesign, staff training, technical support, device-loan programs, and integration of remote data into the electronic health record. These costs may be offset by reduced travel, fewer missed workdays, lower out-of-pocket expenses, more efficient specialist triage, and avoidance of unnecessary in-person referrals (25, 37, 38). Potential reductions in emergency department visits or hospitalizations are clinically important but remain less consistently demonstrated in chronic pain populations. Programs should therefore prospectively measure implementation costs, travel and time savings, no-show rates, downstream utilization, and clinical outcomes rather than assuming that remote care is inherently cost-saving.

### Geographic applicability and generalizability

A primary limitation of this review is geographic bias. Although several cited studies describe chronic pain burden or rural access outside the United States, the intervention evidence, payment environment, integrated health-system examples, and controlled-substance regulatory discussion are predominantly U.S.-based. Telemedicine delivery in Canada, the United Kingdom, Australia, Europe, and other regions may differ because of national health-system organization, reimbursement, privacy law, broadband investment, workforce distribution, and prescribing regulation. The findings and recommendations should therefore be interpreted primarily within the U.S. context and should not be assumed to transfer directly to other countries without local adaptation.

Telemedicine cannot fully replace in-person evaluation or procedural care, but many components of chronic pain management can be delivered effectively when modality selection is matched to clinical risk and patient capability. A hybrid triage model should direct stable follow-up, behavioral treatment, rehabilitation, education, and routine medication review to remote care while reserving in-person assessment for new undifferentiated pain, red flags, unreliable remote examination, interventional procedures, and situations in which opioid safety monitoring cannot be completed adequately. The limitations to telemedicine are summarized below (Table 2).

**Table 2:** Key limitations of telemedicine in chronic pain management.

Limitation Domain	Challenge
Physical Examination	Inability to perform palpation, assess warmth, evaluate allodynia/hyperalgesia, and perform detailed sensory mapping remotely
Range of Motion and Neurological Assessment	Reduced accuracy of certain range-of-motion measurements and complex neurological examinations, particularly in older patients with multiple comorbidities
Interventional Pain Procedures	Telemedicine cannot replace procedures such as epidural steroid injections, nerve blocks, radiofrequency ablation, spinal cord stimulation trials, or implantable therapies
Opioid Monitoring and Safety	Challenges monitoring medication adherence, misuse, substance use disorders, and opioid-related adverse events remotely
Privacy and Confidentiality	Patients may lack a private environment for discussing sensitive topics such as mental health, substance use, and psychosocial stressors
Technology and Connectivity	Limited broadband access, poor internet connectivity, and inadequate access to video-capable devices, particularly in rural communities
Digital Literacy	Difficulty navigating telehealth platforms among elderly patients and individuals with limited technology experience
Patient Preferences	Some patients continue to prefer face-to-face interactions, particularly for complex evaluations and treatment planning
Care Coordination	Difficulty coordinating multidisciplinary pain care across multiple providers and healthcare systems

Table 2. Key limitations of telemedicine in chronic pain management. While telemedicine has expanded access to chronic pain care, particularly in rural and underserved populations, several limitations remain, including challenges related to physical examination, interventional procedure delivery, opioid safety monitoring, privacy, technology access, and patient preferences. Hybrid care models that combine telehealth with strategically timed in-person evaluations may help mitigate many of these limitations while preserving the benefits of improved accessibility and convenience.

A visual summary of the benefits and challenges of telemedicine are shown below (Figure 1).

**Figure 1:**
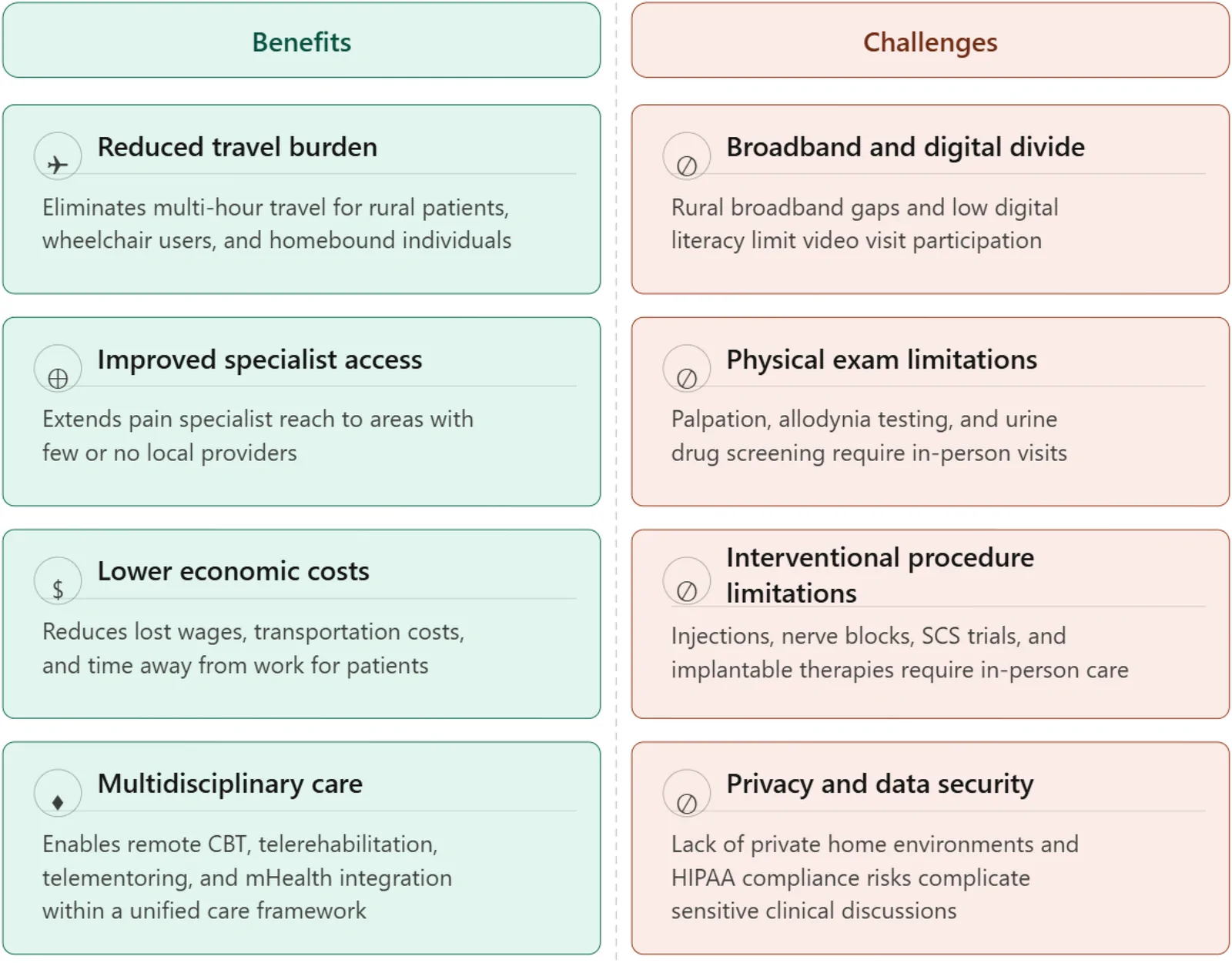
Benefits and challenges of telemedicine for rural chronic pain management.

### Conceptual framework for rural telemedicine success

The proposed Rural Telemedicine Fit Framework (Figure 2) synthesizes why telemedicine succeeds or fails in rural chronic pain care. Success depends on alignment across three interacting domains: patient factors, technology factors, and provider and health-system factors. A strong intervention in one domain may still fail when another domain is inadequate; for example, an evidence-based application offers little value when broadband is unavailable, and high-quality video access cannot compensate for a clinical presentation requiring palpation or urgent neurological examination. The central implementation task is therefore to assess clinical fit and readiness across all three domains and select the lowest-burden modality that preserves diagnostic accuracy, safety, engagement, and equity.

**Figure 2:**
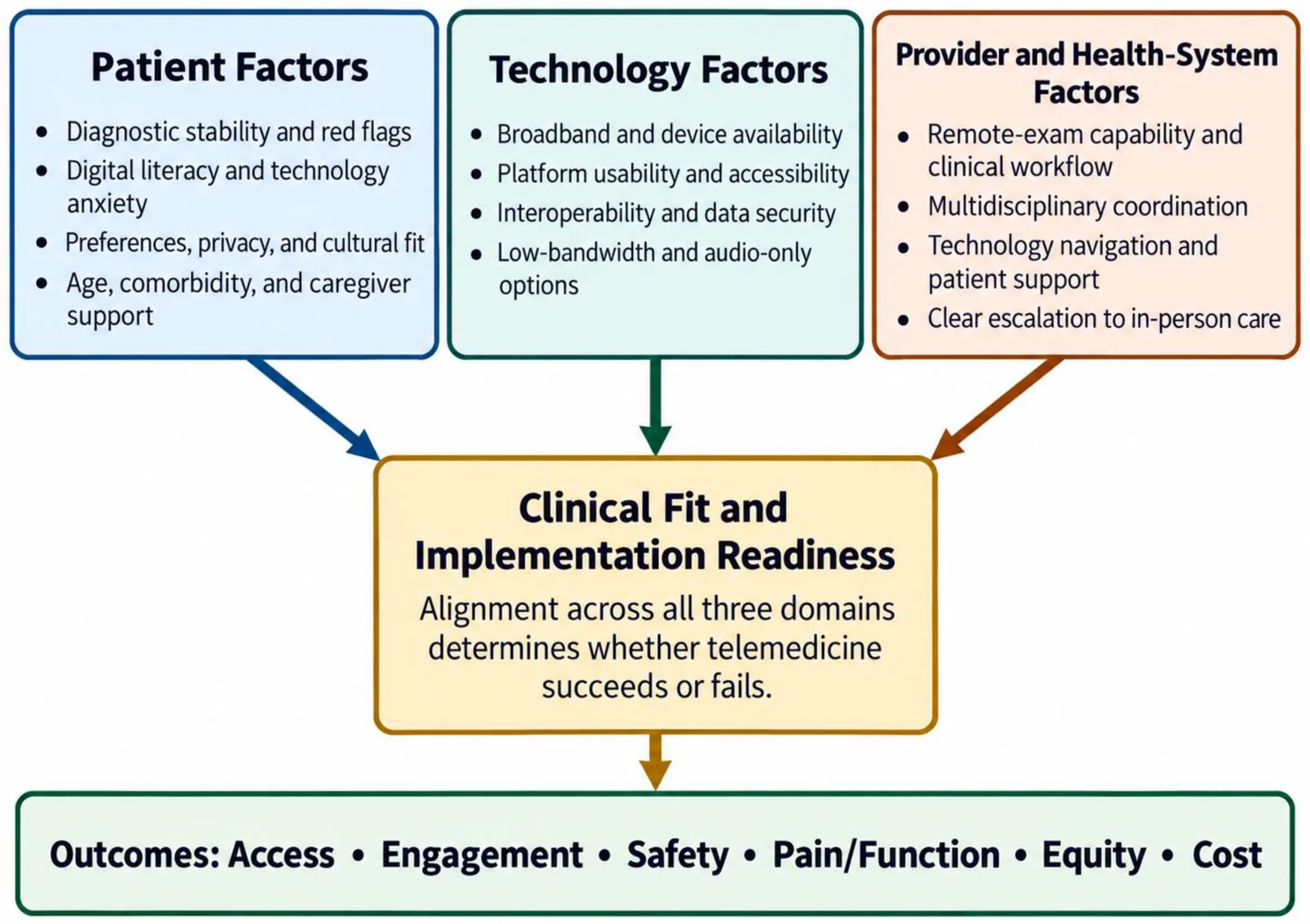
Rural telemedicine fit framework.

### Limitations of the review

The primary limitation of this study is its narrative review design. Unlike a systematic review, study selection was purposive and intended to provide a clinically relevant overview of telemedicine across multiple chronic pain modalities rather than exhaustively identify all available literature. As a result, some relevant studies may not have been included. In addition, the available evidence is heterogeneous with respect to intervention type, patient population, definitions of rurality, comparators, and outcome measures, which limits direct comparison across studies. Several areas are also supported mainly by observational, qualitative, or program-evaluation data, and these findings should therefore be interpreted cautiously.

## Conclusion

Telemedicine is an increasingly evidence-supported strategy for reducing rural disparities in chronic pain care in the United States. Remote CBT and selected telerehabilitation programs have the strongest clinical evidence, whereas specialty video clinics, electronic consultations, VHA programs, and mobile health applications provide important access and engagement benefits but are supported by more heterogeneous evidence. Telemedicine should be treated as a component of multidisciplinary pain care rather than a universal replacement for in-person evaluation.

Health systems seeking to implement or expand rural telemedicine pain services should prioritize the following actions:

1. Use a hybrid triage algorithm that assigns patients to video, audio-only, or in-person care according to diagnostic certainty, red flags, examination needs, procedural requirements, and opioid-monitoring risk.
2. Provide technology-navigation support and low-bandwidth alternatives, including previsit equipment testing, telephone or SMS pathways, interpreter services, caregiver-assisted options, and device-loan programs when feasible.
3. Standardize controlled-substance workflows with current DEA/HHS policy review, prescription drug monitoring, clearly documented risk assessment, toxicology testing when clinically indicated, and reliable access to in-person evaluation.
4. Integrate remote CBT, telerehabilitation, and electronic specialist consultation into a coordinated multidisciplinary pathway rather than offering isolated video visits without rehabilitation or behavioral support.
5. Measure equity and value prospectively by stratifying engagement and outcomes by rurality, age, broadband and device access, disability, and preferred modality while also tracking travel savings, no-show rates, implementation costs, and downstream healthcare utilization.

The evidence and regulatory framework summarized here are primarily U.S.-based and may not translate directly to other countries. Within the U.S. context, programs that combine patient choice, technology support, structured hybrid triage, multidisciplinary treatment, and explicit safety escalation are most likely to improve access without sacrificing clinical quality or widening the digital divide.
